# Treatment of Perforation Peritonitis

**Published:** 1894-10-27

**Authors:** 


### TREATMENT OF PERFORATION PERITONITIS.

The discussion at the Medical Society on Monday on "Ulceration of the Duodenum," together with that at Bristol on "The Operative Treatment of Perforative Ulcers of the Stomach and Intestines," puts us in possession of the actual state of opinion at the present moment in regard to the treatment of what one may term perforation peritonitis, or the peritonitis arising from the effusion of the intestinal contents.

It is unnecessary to point out the importance of accurate diagnosis. Unfortunately, it is often quite impossible until the catastrophe of perforation has occurred. For although sometimes there is a history of preceding symptoms fairly distinctive of one or other of the conditions which most commonly lead to perforation, it so often happens that the mischief occurs without warning that if good is to be done we must be prepared to operate without waiting for exact knowledge of the seat of the rupture.

With the sudden onset, then, of the symptoms of perforation, what are we to do? The first thing to remember is that fouling of the peritoneum by perforation of a gastric or duodenal ulcer is almost universally followed by death, and that desperate remedies, if they do but occasionally cure, are justifiable in desperate diseases. The uniform fatality of expectancy is the justification of operative treatment. This has two aims—(1) the cleansing of the peritoneum, for it is the peritonitis that kills the patient; and (2) the closure of the opening through which the effusion takes place.

The cloud of doubt hanging over the question of the locality of the ulcer, and hence of the possibility of getting at it would, however, make the operation of laparotomy in such cases one of the most uncertain ventures of surgery if the closure of the ulcer were the only aim of the operation. This, however, is not the case. Even if nothing more is done than mere cleansing something is gained. In a considerable number of cases, however, the perforation can be found and can be closed, and in some a cure has resulted from this proceeding.

There can then be no doubt what should be done—a laparotomy should be performed. If the effusion in the peritoneum should be acid and free from feculent odour the presumption is very strong that it comes either from the stomach or duodenum. Search then should be made, and if an ulcer can be found it should if possible be drawn out and carefully closed by means of a Lembert's suture. If the stomach should be found full of food it should first be washed out, and in any case it may be found advantageous to do this if there is time and the patient can bear it. The peritoneum should then be cleansed with the greatest care, which may partly be done by sponges or by folds of aseptic gauze, but principally by flushing with boiled water of a temperature of 110 degrees Fahr. to 112 degrees Fahr., to which salt may be added in the proportion of ʒi to a pint. The greatest care must be taken in this part of the operation, for no amount of accuracy in closing the ulcer will save the patient if the peritoneum be left with pouches full of septic material. When the wound is closed it is generally advantageous to insert a drainage tube leading down into the pelvis, discharging, if necessary, through a separate opening made above the pubes.

What to do when it is impossible to close, or may be even to get at, the ulcer is a grave question, with an answer to which the surgeon must be ready. If the ulcer should be situated close to the abdominal wound, or so near that it can be fixed to it without strain, so as to secure a gastric fistula, this would be a good plan, but the cases in which this can be done are the very cases in which the ulcer can usually be closed by suture. If, however, it cannot be drawn up to the abdominal wound in this way the best plan probably is, after thorough cleansing of the peritoneum, to pass one end of a tube into the ulcer and bring the other end out of the wound, packing it well round with iodoform gauze all the way down. It must never be forgotten, however, that if in a case of perforative peritonitis imagined to arise from the vermiform appendix no disease can be found there, search should be made at the stomach and duodenum; and if, on the contrary, when these parts are thought to be the locality involved they should prove intact, search must be made about the cœcum; and whichever lesion is dealt with, above all things the peritoneum must be made clean.

